# Supplementary material for: Are carbon emissions trading and green financial instruments synergistic? -Comprehensive quantitative research based on content analysis
Source: PLoS One. 2024 Mar 7;19(3):e0298601. doi: 10.1371/journal.pone.0298601 (PMC10919601; doi:10.1371/journal.pone.0298601)
Supplement: S1 Data — (ZIP) [file pone.0298601.s001.zip › Data/地级市Z_F工作报告.zip!/┴╔─■/╠·┴δ╩╨/└┤╫╘╣1⁄2╓┌║┼í╛┬φ┐╦╩2╛▌═°í┐_2005.docx]

各位代表：

现在，我代表市人民政府向大会报告工作，请予审议，并请市政协委员和其他列席会议的同志提出意见。

一、2004年工作的回顾

过去的一年，是全市各项工作不断开创新局面的一年。我们在省委、省政府和市委的正确领导下，团结和带领全市各族人民，坚持以邓小平理论和“三个代表”重要思想为指导，深入贯彻党的十六大和十六届三中、四中全会精神，树立和落实科学的发展观，全力实施开放立市、工业强市、城乡统筹、科教兴市和全民创业“五大战略”，积极稳妥地解决经济社会发展中的突出矛盾和问题，全面完成了市五届人大一次会议确定的各项任务，国民经济和社会事业取得了比较显著的成就，提前一年实现了“十五”计划的主要奋斗目标。全市GDP实现216．1亿元，增长17％，其中第一产业增加值57．6亿元，第二产业增加值89．7亿元，第三产业增加值68．8亿元，分别增长11％、26．3％和11．7％。全社会固定资产投资完成78．7亿元，增长23．3％。社会消费品零售额实现85．4亿元，增长15．8％。地方财政一般预算收入完成10．9亿元，一般预算支出27亿元，分别增长16．1％和11％。农民人均纯收入3482元，城镇居民人均可支配收入5724元，分别增长11％和2．1％。金融机构存款余额238．2亿元，贷款余额226．6亿元，分别比年初增加29．1亿元和21．2亿元。人口自然增长率控制在3．31‰。

一年来，我们紧紧围绕强市富民目标，突出重点，真抓实干，主要做了以下10个方面的工作。

1．继续加大招商引资力度，对外开放取得丰硕成果

坚持开放立市战略，深入开展“项目年”活动，精心组织国内外招商活动，特别是成功地举办了第二届国际民间艺术节。全市共引进域外投资项目496个，其中超亿元大项目13个；到位资金80．4亿元，其中生产加工型项目到位资金占84％；新批外商投资企业53家，合同外资额2．4亿美元，增长9％；实际利用外资8109万美元，增长6％，招商引资的水平和质量上了一个新台阶。紧紧抓住国家振兴东北老工业基地和省委、省政府加快辽西北开发的历史机遇，争取到国家和省支持的各种资金17．58亿元，其中用于基本建设和事业发展的资金6．86亿元。外贸出口实现8125万美元，增长40．1％；外派劳务2200人，增长25．7％。组建了铁岭出入境检验检疫局，极大地方便了进出口企业和出国出境人员。开发区保持快速发展的好势头。旅游业持续升温，实现旅游总收入7．9亿元，增长27．4％。

2．突出工业强市的主导地位，工业经济保持快速增长的强劲势头

大力营造工业强市的浓厚氛围，坚持改革、改组、改造相结合，切实加强工业园区建设，加大了对工业发展的服务扶持力度，使全市工业继续保持较高的增长幅度。全市规模以上企业新增85户，总数达到350户；全部工业总产值突破200亿元，规模以上工业完成工业总产值165．9亿元，增长31％；实现工业增加值61．8亿元，增长29．9％，工业增加值占GDP的比重提高了3．4个百分点；实现利税1 3．2亿元，增长10．5％。有115户国有企业实施了各种形式的产权制度改革，改革面达93．5％。国有资产监督管理体制得到初步理顺，第一批51户国有及国有控股企业划归市国资委管理。铁西工业区开发改造取得较大进展，已经有5个项目入驻；制定了橡胶产业园规划和建设方案并开始对外招商；启动建设44个县乡工业园区，落户企业已达820户，成为当地经济发展新的增长点。技术改造步伐加快，全年共完成技改投资14．8亿元，开发新产品160项，实现新产品产值12亿元。积极推进名牌战略，共有12个产品获省级名牌，20个产品获市级名牌。

3．坚定不移地推进结构调整，农业农村经济实现了新突破

全面贯彻中央一号文件精神，认真落实粮食直补、土地延包等政策，积极稳妥地进行农村税费等项改革，调动了农民的生产积极性，粮食总产量达31．7亿公斤，创历史最好水平。坚持用工业理念谋划农业，农业产业化取得新进展，新上投资1000万元以上的产业化龙头项目12个，全市固定资产投资1000万元以上的产业化龙头企业已发展到30户，其中国家级和省级5户，全市农产品加工总量达到230万吨，形成具有一定规模的区域性产业化基地140多个，总面积发展到300万亩，定单农业已发展到210万亩．坚定不移地推进农业农村经济结构调整，大力实施“畜牧业倍增计划”，新建村外畜牧小区50个，畜牧业产值实现62亿元，增长15．5％，占农业总产值的比重达到46．2％；种植业结构和农产品结构进一步优化，专用粮食、蔬菜和经济作物种植面积发展到538万亩，无公害农产品认证达到50个，获得绿色食品标志产品达到20个。农业基础设施和生态建设得到加强，共动用土石方6344万立方米，完成农建重点工程280多项，治理水土流失面积17．6万亩，植树31万亩、8468万株。农业标准化、扶贫开发、农村市场建设等工作也都取得了新成绩。

4．营造全民创业的浓厚氛围，民营经济跨上新台阶

制定出台了鼓励民营经济发展的优惠政策，采取有力措施改善民营经济发展环境，努力营造全民创业的浓厚氛围，促进了民营经济的快速健康发展。全市民营企业和个体工商户发展到12．4万户，其中新增0．9万户，从业人员44万人。实现增加值90亿元，增长28．6％。实缴税金9亿元，增长38．5％。出口交货值实现5．9亿元，增长20％。新上投资500万元以上项目143个，完成固定资产投资42．5亿元，增长52．9％。民营经济在全市经济发展中正在发挥越来越大的作用。

5．加大城乡基础设施建设力度，城镇化步伐进一步加快

加快了中心城市建设步伐，新建、改建南马路等城市道路10条，开工建设了南柳游园，完成了银北热源厂二期工程和铁岭垃圾处理厂前期准备工作，解决较高标准的社区办公用房19处．新建和改造了排水、煤气管网，城市的基础设施日趋完善．实施了较大规模的城市绿化，亮化工程，特别是南马路亮化工程成为市区又一景观。中心城区开发建设住宅42万平方米，改造棚户区占地面积51．6万平方米．加大了城市管理的力度，脏乱差现象有较大改变。加大土地经营力度，全市经营性用地使用权全部实行招拍挂，市本级获得直接收益首次突破1亿元，同时追缴历年拖欠的土地出让金3185万元。规范土地市场秩序，查处违法用地案件163件，清退土地4．6公顷，偿还拖欠农民征地补偿费1365万元。完成了15个建制镇的总体规划和专业规划的修编，村镇基础设施和村容镇貌得到较大改观．交通建设又有新进展，完成村通油路工程421公里，建设桥梁86座，102线平顶堡至毛家店104公里二级路改扩建工程竣工通车。切实加强环境保护工作，对重点污染源进行了专项整治，积极推行集中供热，人居环境得到明显改善。

6．全力实施“一号工程”，社保和再就业工作取得显著成效

社会保障体系进一步健全，各类保险总参保人数已达117．4万人，其中新增22．4万人，养老保险个人帐户基本做实。千方百计筹集资金4亿多元，确保了离退休人员养老金的按时发放。失业保险实现市级统筹，为近7万名享受失业保险待遇人员发放失业金1．5亿元。为全市414户无力参保的集体企业1．2万名退休职工按低保标准发放了生活费。全市农垦企业在职及退休职工全部纳入社会保障范畴，离退休金及遗属生活费全部实现社会化发放。市直困难企业、并轨人员和灵活就业人员5．7万人已纳入医疗保险。偿还并轨人员债务工作取得重大进展，偿债率达95％。实行城乡就业统筹，加强职业培训基地建设，组织34家培训学校，培训下岗失业人员2．3万人、农民1000人。认真落实就业和再就业优惠政策，共发放小额贷款756万元，实现实名制就业5．7万人。筹集扶贫帮困款物合计3600万元，帮扶困难户3万户、7．2万人，为城市低保户减免取暖费和为城市平房户发放取暖补助共计248万元，为农村特困户建房150户，兴建9所区域性中心敬老院。城乡最低生活保障对象达9．1万人，城市保障金实际发放标准人均每月提高10元。此外，还提高了老工商业者的养老保险待遇。

7．充分发挥科技和人才的前导作用，科技和人才工作出现新局面

召开了全市科技大会和人才工作会议，科教兴市和人才强市意识进一步增强。全面启动科技创新工程，加强了科技服务平台建设，安排市本级重大科技计划项目22项，专利申请量完成484件，铁丰大豆29号新品种选育及应用获省科技进步三等奖。引进农业新品种50多个，推广农业新技术20多项。新增民营科技企业60户，新发展高新技术企业2户，实现高新技术产值21．5亿元。加强了与中科院沈阳分院等科研院所的科技交流与合作，签订科技合作协议33个。人才工作出现多年未有的好局面，接收安置具有学士学位的应届本科毕业生450人，在省内外举办各种形式的招聘会216次，6042人与用人单位达成协议。加大了人才培养力度，在法国南希、上海芦湾区等地建立了人才培训基地。执行引智项目28项，新征集引智项目66项，铁岭农科院被评为省首家国家农业引智示范基地。“一村一名大学生”计划取得新进展，今年又选调506名农村优秀青年到高校脱产深造。

8．更加注重经济社会协调发展，各项社会事业取得新的成就

教育改革与发展稳步推进，进一步落实了“以县为主”的教育管理体制，农村公办教师工资全部实现县级统筹，工资发放标准有了大幅度的提高，基本解决了前3年的工资拖欠。我市发起的“春晓工程”在全省推广，调整撤并农村小学234所，新建学校20所，改造危房近10万平方米。完成6所农村高中建设，高中阶段扩招1786人。职业教育健康发展，高等教育办学水平有新的提高，教师队伍结构得到改善。文化事业更加繁荣，成功地举办了第二届国际民间艺术节暨第二届赵本山杯二人转大赛，积累了按市场规律发展文化产业的经验，进一步提高了铁岭在海内外的知名度。开工建设新图书馆，修复了银冈书院。积极推进卫生监督与疾病预防控制体制改革，大力加强了以国债建设项目为重点的疾病预防控制体系、医疗救治体系建设。加大了公共卫生监管力度，深入开展了以城乡环境卫生综合整治为主要内容的爱国卫生运动。体育战线再传佳讯，我市输送的运动员李卓、孙福明分获雅典奥运会银牌和铜牌。广播电视事业快速发展，广播电视大厦已完成主体工程，改建了电台调频发射塔，进一步扩大了广播覆盖面。低生育水平持续稳定，出生性别比得到控制，计划生育技术服务水平有很大提高。深入开展“手拉手”帮扶民族乡镇活动，促进了少数民族地区经济的发展。初步建立了依法管理宗教事务的网络体系和预警机制，维护了信教群体的稳定。启动了创建文明城市工程，精神文明建设和未成年人思想道德建设得到加强。“双拥”工作取得新进展，进入省级“双拥模范城”行列。

9．深入扎实地开展“环境建设年”、“安全年”活动，经济发展环境得到进一步改善

市政府与48个部门和单位签订了纠风和软环境建设责任状，调查处理了一批影响软环境的案件。深化“行风热线”活动，市长、各位副市长相继走进直播间，得到上级有关部门的充分肯定和人民群众的广泛好评。强化了公共行政服务中心的职能，进一步提高了办事效率。开展了安全生产大检查和专项整治活动，排查、整改了一大批事故隐患，建立健全了安全生产监督管理机构，安全生产长效机制逐步完善。加强了社会治安综合治理，完成了公安警务体制改革，开展了集中侦破杀人案件等专项活动，制定了突发性事件应急预案，认真受理人民群众来信来访和市民投诉，解决了大量关系群众切身利益的实际问题，全市安全事故率、交通事故率、群体事件发案率、刑事案件发案率4项指标均保持较低水平。加强社会信用体系建设，开展了以打击制售假冒伪劣产品为重点的整顿市场秩序专项活动，进一步规范了市场环境。

10．切实加强政府自身建设，行政能力和水平有了新的提高

认真贯彻落实《行政许可法》，对行政许可依据、许可主体和许可项目开展了深入细致的“三清”工作，废止和修改包含行政许可内容的文件13件。进一步完善行政执法责任制和评议考核制，加大法制监督力度，依法做好行政复议工作，提高了依法行政水平。积极稳妥地推进政府机构改革，进一步理顺了部门职责，进一步削减了行政审批事项，提高了行政效能。建立了政府领导与人民群众沟通对话、重大事项新闻发布、涉及群众切身利益事项举行听证三项制度，密切了政府与人民群众的关系，加强了与民主党派的联系，推进了科学行政、民主行政。落实廉政建设责任制，认真遵守领导干部廉洁自律的各项规章制度，严肃查处各种违法违纪案件，加大审计监督工作力度，注重从源头上防范和治理腐败，教育、制度、监督并重的反腐败工作格局正在形成。

统计、物价、工商、国家安全、司法、商业、粮食、供销、药品监督、民防、农机、烟草、外事、侨务、台办、档案、气象、地震、电业、邮政、电信、仲裁，市志、老龄、残联等工作也都取得了新成绩。

各位代表，一年来各项成就的取得，是省委、省政府和市委正确领导的结果，是市人大、市政协依法监督、民主监督与大力支持的结果，是全市人民精诚团结、奋力拼搏的结果。在此，我代表市人民政府，向辛勤工作在各条战线上的全市各族人民，向热情支持政府工作的各民主党派、工商联、无党派爱国人士以及社会各界人士，向解放军驻铁部队指战员和武警官兵、中省直驻铁单位，向广大域外投资客商，向全国各地以各种方式支持家乡建设的铁岭同乡，向所有关心支持铁岭改革开放和现代化建设的海内外朋友表示衷心的感谢并致以崇高的敬意！

在看到成绩的同时，我们也清醒地看到，在我市经济发展的关键阶段，还存在不少长期积累、制约全局的深层次矛盾和问题，我们的工作与上级和全市人民的要求还有不少差距。一是经济总量小，工业化、城镇化水平低，经济结构不尽合理，经济发展缺乏大项目的支撑，主导产业尚未形成，经济运行质量还不够高，县乡财政运行困难。二是“三农”问题仍是我市发展中的难点和重点，农业基础设施薄弱，农业综合生产能力不强，农民增收渠道还不宽。三是经济和社会事业发展不够协调，各项社会事业特别是农村社会事业发展滞后的局面还没有根本改变。四是经济和社会发展中还存在不少矛盾和困难，就业和社保压力大。五是政府自身建设与“三个代表”和提高执政能力的要求还存在不适应、不符合的问题，经济和社会发展环境还有待于进一步改善。

二、2005年工作的指导思想和主要预期目标

2005年是全面实现“十五”计划目标、为“十一五”发展奠定基础的重要一年。我们要以邓小平理论和“三个代表”重要思想为指导，认真贯彻党的十六大和十六届三中、四中全会精神，用科学发展观统领经济社会发展全局，坚持发展第一要务，积极稳妥地推进各项改革，进一步扩大对外开放，大力调整经济结构，切实转变经济增长方式，优化经济发展环境，深入实施“五大战略”，努力做大中心城市，着力建设“两大基地”，重点培育“五大主导产业”，确保经济发展规模、速度、效益同步增长，加快发展各项社会事业，构建和谐铁岭，实现物质文明、政治文明和精神文明全面协调发展。

按照上述指导思想，在政府工作实践中要做到“八个必须”：1．必须保持较快的发展速度。面对不进则退、慢进亦退的发展形势，在注重调整优化经济结构、转变经济增长方式的同时，要继续保持较高的增长速度，做到经济发展规模不减、速度不减、效益不减。2．必须坚持经济社会协调发展。坚持以经济建设为中心不动摇，同时要注重统筹经济和社会协调发展，加快解决社会事业特别是农村事业发展滞后的问题。3．必须坚持区域经济协调发展。做大做强中心城市，加快县城向中小城市迈进步伐，发展辽北中小城市群，大力推进城镇化进程，形成中心突出、梯次推进、以城带乡、协调发展的区域经济新格局。4．必须坚持以人为本。把维护好、实现好、发展好人民群众的根本利益体现在各项工作部署中，落实到经济社会发展的各个方面，让发展和改革的成果惠及广大人民群众。5．必须坚持深化改革。有步骤、有重点地推进各项改革，着力解决制约经济和社会发展的体制性、机制性矛盾，力争在一些重点领域和重点环节的改革上取得新突破。6．必须坚持扩大开放。坚定不移地实施开放立市战略，在更大范围、更广领域、更高层次上推进对外开放，以开放促改革、促调整、促发展。7．必须坚持全民创业战略。进一步营造全民创业氛围，加快民营经济发展。同时要进一步搞活国有、集体企业，努力提高企业竞争力。8．必须坚持科学行政、民主行政、依法行政。以解决“不适应、不符合”问题为切入点，切实加强政府自身建设，努力提高各级政府的执政能力，树立为民形象，打造亲民政府。

2005年国民经济和社会发展主要预期目标是：

保持国民经济快速健康发展。GDP实现255亿元，增长18％，人均GDP实现1000美元。全社会固定资产投资完成95亿元，增长20％。

着力增强财政实力。地方财政一般预算收入10．5亿元，比上年预算增长18％；一般预算支出安排22．9亿元，比上年预算增长23％。

努力提高人民生活水平。社会消费品零售总额实现99亿元，增长16％。城镇居民人均可支配收入6180元，增长8％；农民人均纯收入3800元，增长9％。

统筹经济社会协调发展。实现就业4．3万人。人口自然增长率控制在3‰左右，城镇登记失业率控制在5．9％以内。

三、以进一步调整优化经济结构为重点，促进国民经济快速健康发展

今年的经济工作，要重点抓好以下7个方面：

(一)以项目招商为重点，全面提高对外开放水平

进一步明确招商引资的重点领域，吸引域外资金投向“两大基地”、“五大主导产业”以及城市基础设施、社会事业等领域，突出抓好大项目、高附加值项目和利用外资项目。坚持引资与引智相结合、走出去与请进来相结合，采取更加务实、更加灵活的招商方式，积极推动招商活动由政府主导型向企业自主型转变。突出招商的重点国家和国内的重点区域，建立大项目协调机制和全程跟踪服务责任制，努力提高项目的成功率、资金到位率和开工率。继续开展“项目年”活动，特别是要抓紧做好项目的规划、论证、包装和上报工作，积极向上争取项目和资金。加强对外经贸队伍建设，培养和引进更多的外经外贸人才。全年招商引资实际到位资金额60亿元，实际利用外资9730万美元，增长20％。

千方百计扩大外贸出口。扶持企业积极开拓国际市场，积极引进出口主体，发展出口代理，组建出口企业协会，全面提高出口能力。加强出口基地建设，整合出口资源，优化出口商品结构，积极开拓非洲、拉美、东欧等新兴国际市场。鼓励和扶持有条件的企业到境外投资办厂，带动产品出口。积极向上沟通，筹建铁岭海关。用好、用足上级扶持外贸出口的各项政策，加大出口扶持力度，全年完成外贸出口9750万美元，增长20％；外派劳务2640人，增长20％。

(二)做大做强工业经济，努力提高工业化水平

科学搞好产业定位。加强产业调整的研究和规划工作，充分利用我市煤炭、农产品等资源优势和毗邻辽宁中部城市群原材料生产基地、装备制造业基地的区位优势，着力建设能源、优质农产品生产加工“两大基地”，大力发展煤炭电力、食品加工、机械和汽车零部件配套、建材、纺织造纸等“五大主导产业”，尽快形成铁岭产业特色，使其成为我市的优势产业。此外，通过上级支持、招商引资等途径，争取新上昌北煤矿、玉米秸秆造纸、煤矸石发电等一批依托资源优势的大项目，促进全市工业的发展，提高工业化水平。

做大做强一批具有较强竞争力的骨干企业。支持铁煤集团在稳产高产的同时大力发展非煤产业，加快清河电厂“以大代小”改造步伐，开工建设铁岭电厂二期工程，重点抓好华晨、大成等33户产值超亿元的骨干企业，培育陆平机器等5户产值超10亿元的企业集团，增加规模以上企业100户。大力实施名牌、品牌战略，走抓品牌、上项目、壮企业、兴产业的路子。加大对企业扶持力度，建立健全贷款担保体系，组建中小企业协会和建材、食品等行业协会，积极帮助具备条件的股份制企业上市，为企业发展提供有利条件。

加快开发区和工业园区建设，培育产业集群。进一步加强开发区基础设施建设，切实发挥其先导和示范作用。加快铁西工业区开发改造步伐，抓紧启动橡胶科技园建设，尽快把铁西建成新兴的工业区。抓好县乡工业园区建设，争取5个园区产值超5亿元。自觉接受沈阳的经济辐射，全面加强两市间的经济合作，加快构建沈铁工业走廊。

深化国有企业改革。已经实施产权制度改革的企业重在加快现代企业制度建设，完善法人治理结构。未改制企业依法解除租赁企业的租赁合同，实行资产优化重组。对空壳企业，实行债务统一回购、资产和收益统一管理。今年要基本完成国有企业的产权制度改革。完善国有资产监督管理体制，建立产权交易机构，加强对国有企业经营业绩考评，建立符合市场经济要求的企业经营者管理体制和企业人力资源配置机制，提升企业家队伍素质。

(三)以增加农民收入为目标，加快农业农村经济结构调整和农业产业化进程

坚持城乡统筹，继续把解决“三农”问题作为工作重点．积极推进农业和农村经济结构战略性调整，努力提高农业综合生产能力。深化农业内部结构调整，全面实施“畜牧业倍增计划”，新建村外畜牧小区50个，实现畜牧业产值68亿元，增长9．7％，争取率先建成畜牧强市。进一步抓好以蔬菜和高效经济作物为重点的种植业结构调整，加快推进西部旱作节水经济作物带、东部山区特色产业带和中部优质专用粮油、水果蔬菜产业带建设。加快农产品结构调整，实施农产品市场准入制度，促进农产品生产向优质化方向发展，大力打造品牌农业，提高我市农产品的市场竞争力。全市注册农产品品牌20个，绿色食品标志达到40个，无公害农产品认证达到100个。加快调整农村劳动力就业结构，加强对农民工的职业技能培训，大力发展劳务经济，提高农民劳务输出的组织化程度，促进劳动力向非农产业转移。

加快农业产业化步伐。进一步落实和完善扶持农业产业化龙头企业的政策和措施，尽快把北绿、依露、雨润等20户现有龙头企业做大做强。围绕肉鸡、生猪、肉牛、乳制品等7大重点产业，通过招商引资、吸引民间资本等渠道，新增固定资产投资1000万元以上的龙头企业10户，其中超亿元企业5户。加强农产品基地建设。以发展无公害、绿色、有机农产品为重点，以区域化、规模化为方向，重点规划建设玉米、蔬菜、肉牛、奶牛等重点产业的生产基地，使基地成为工业的第一车间，全市基地面积达到330万亩，带动农户30万户。引导龙头企业与基地之间建立稳定的合同关系和紧密的利益联结机制，形成真正的利益共同体。充分发挥各类农业行业协会、农业生产专业合作组织以及中介组织、农村经纪人的作用，加强信息服务网络建设，培育和完善农产品流通市场。

切实加强农业保障体系建设。加大资金投入力度，着力完善防洪、排涝、灌溉、水土保持“四大工程体系”，完成农田基本建设土石方8000万立方米，新增水利工程面积65万亩。加强以林业为主的农业生态环境建设，全市完成造林面积20万亩，植树5000万株。进一步提高农业机械化水平，农机总动力达到130万千瓦，农业综合机械化水平达到41％。加强农业良种体系、科技推广体系、动植物保护体系等农业保障体系建设，努力提高为“三农”服务的保障能力。

认真落实农村各项法规和政策。全面落实农业税减免和各项直补政策，促进农民增收。继续抓好农村税费改革，落实转移支付等政策，推进乡镇事业单位特别是农村“五站”改革，积极化解村级债务，切实减轻农民负担。认真贯彻《土地承包法》，完善第二轮土地延包工作。完善规范征地程序，保障失地农民的合法权益。

(四)改造提升传统第三产业，大力发展现代服务业

引进国内外知名商家，积极发展现代流通业态，培育大型物流集团。延伸流通业触角，探索农副产品和日用工业品双向流通的网络体系。逐步扩大消费信贷，拓展新的消费领域。改造商业设施，打击制售假冒伪劣行为，创造舒适、诚信的消费环境。深化粮食流通体制改革，放开粮食市场，千方百计解决农民卖粮难问题。大力发展农村客运业，基本实现村村通客车。加快旅游资源开发、整合步伐，加强与省内外的旅游合作，大力开发国内外旅游市场，打造生态游、小品之乡游等旅游品牌，争创全国优秀旅游城市，进一步做大做强旅游产业，实现旅游业总收入10亿元，增长26．6％。加快发展并规范房地产业，中心城区开发住宅46万平方米。大力发展社区服务、中介服务等现代服务业，积极培育土地、劳动力、资本、技术等生产要素市场，提升第三产业的档次和水准。

(五)激发全民创业热情，促进民营经济大发展、快发展

国有经济以“两电一煤”为主，地方经济以民营为主，培育壮大地工型民营经济，扶持转制型民营经济，大力引进外来型民营经济，促进民营经济提速发展。坚持政治上放心放胆、发展上放开放手、经营上放宽放活原则，旗帜鲜明地鼓励创业者，使创业文化成为全市的主流文化，创业精神成为铁岭精神的内核。进一步清理与民营经济发展不相适应的各种文件规定，消除市场限制和政策歧视，切实降低准入门槛，成立民营经济权益维护中心和法律顾问机构，坚决清理和制止“三乱”，为民营经济发展创造宽松的外部环境。鼓励民营企业并购国有和集体企业，允许民营经济参与城乡基础设施建设，投资城镇公用事业，大力支持民营资本投资现代服务业。引导民营企业建立现代企业制度，推进科技创新、机制创新、管理创新，促使民营企业家开阔视野，更新观念，加快提升综合素质。开展“争创百强民营企业”活动，全市民营企业和个体工商户新增5000户，从业人员新增1万人，实现增加值115亿元，增长27％；实缴税金10．4亿元，增长16％。

(六)努力增加财政收入，充分发挥财税金融对地方经济发展的促进作用

大力组织财税收入。加强重点税源和税种的征管，加大税收稽查力度，挖掘增收潜力，大力清理欠税，努力做到应收尽收。通过强化土地收益管理、整合和运营国有资产等措施，盘活存量资产，增加财政收入。进一步加强非税收入管理，严格执行“收支两条线”规定，增强政府可调控财力。调整优化支出结构，大力压缩一般性支出，确保公教人员工资按时足额发放和社会保障资金需要，重点保证农业、教育、科技等法定支出。加强对财政性资金运行全过程的审计监督，严肃财经纪律。

加强企业和社会诚信建设，密切银企联系，搞好项目对接，积极争取上级金融机构授权授信，大力支持重点工业项目、农业产业化项目、城市基础设施建设项目和新兴第三产业项目，充分发挥金融对地方发展的支撑作用。完成城市信用社和农村信用社的改革，发挥其在发展民营经济和农村经济上的重要作用。强化金融监管工作，防范和化解金融风险。

(七)深入开展“环境建设年”活动，进一步优化经济发展环境

以行风建设为重点，进一步优化政务环境。认真开展行风评议活动，把评议结果与部门综合位次考评挂钩。认真受理域外客商和广大群众对行风及“三乱”问题的投诉和举报，严肃查处影响软环境的各种违纪行为并公开曝光，真正架起软环境“高压线”。继续办好《行风热线》，解决人民群众关心关注的热点难点问题。完善公共行政服务中心的职能和运行机制，创新工作流程，减少审批环节，研究和实行相对集中行政许可权、并联审批等制度，为域外客商和广大人民群众提供优质、便捷的服务。切实落实招商引资、发展民营经济等各项优惠政策，着力解决“中梗阻”问题，继续清理与发展市场经济和wT0规则相悖的文件规定，进一步优化政策环境。加强社会信用体系建设，规范各类中介机构，维护正当的竞争秩序，严厉打击制售假冒伪劣行为，创建“诚信铁岭”，进一步优化市场环境，把铁岭打造成服务高地、成本洼地和发财宝地。

四、做大做强中心城市，加快发展县域经济，加速推进城镇化进程

按照“做优城市规划，做强城市产业，搞活城市经营，完善城市功能，优化城市环境”的总体思路，做大中心城区，做强中心城市，充分发挥中心城市对区域经济发展的辐射和带动作用。一是按照建成大城市的目标，调整和完善中心城区总体规划，扩大配置各种资源的空间，启动建设柴河新区，推进“山水城市”建设。二是科学搞好产业规划，重点抓好开发区建设和铁西工业区开发改造，加快现代服务业的发展，不断壮大城市的产业支撑。三是加快完善城市功能。今年重点抓好10项工程：1．改造棚户区25万平方米、居民小区5个。2．搞好8条主次干道和14条小街小巷路面改造，解决30条小街小巷亮化问题。3．建设市民广场、银北游园，完善南柳游园。4．新建、改建36座公共厕所。5．改造市区排水。6．开工建设铁岭垃圾处理厂。7．加快城区集中供热联网步伐。8．改造城区北出口。9．实施城区绿化工程，市区植树20万株，新增绿地20公顷，新增公共绿地5公顷。10．开工建设金山购物广场、龙首市场二期精品城、电子商业大厦、房地产交易市场4个区域性商贸中心。四是进一步加大城市环境综合整治力度。提高卫生保洁标准，继续推进亮化工程，提升市容环境，加大城市管理综合执法力度，使城市管理走上法制化、经常化、规范化的轨道。五是强化经营城市理念，盘活做大城市资本。建立健全土地储备制度，严格实行经营性土地使用权招标拍卖，严格建筑市场管理，加大公用事业改革力度，逐步建立投资多元化、运作市场化、经营企业化、服务规范化的公用事业体系，加快各种城市资产走向市场化的步伐。

坚持“一主三化”方针，加快县域经济发展。一是加快县城和以新台子、八面城为重点的中心集镇的发展。搞好采煤沉陷区治理，积极推进调兵山市城乡一体化进程。制定并实施开原、清河城市发展建设一体化规划。加快铁岭县城向新台子搬迁步伐，加强新台子工业园区建设，发挥其在沈铁经济合作中的联结点作用．努力提升和完善昌图、西丰县城的功能，增强对县域经济的拉动力。二是加快县乡工业园区建设，充分发挥资源优势，发展各具特色的优势产业和支柱企业，注重产业集群效应，走出特色经济之路。三是加大对县域经济的扶持力度。进一步下放经济管理权限，调整市、县两级财政分配关系，市、县共享税种增量部分从今年起全部返还县级，落实取消县级部分专项上解、减少配套资金等政策。加大对县域经济的投入力度，建立县域经济财政贴息基金，采取以奖代补、增加困难县乡转移支付补助等措施，调动和保护县乡发展经济和组织收入的积极性。四是加强县域之间交通基础设施建设。完成昌法线45公里二级路路基改造工程，改造公路干线路基133公里，改建黑色路面72公里、桥梁17座。

着力推进城镇化进程。以铁岭中心城市为中心，以县城和新台子、八面城等中心集镇为重点， 以市域主要交通干线为轴线，选择12个重点镇优先发展，尽快形成互相补充、梯次推进、浑然一体、共同发展的小城镇空间布局。一是加快完善小城镇建设规划。加大资金扶持力度，在未来2年内全部完成小城镇规划的第二轮修编，切实发挥小城镇建设总体规划的宏观调控作用。二是在试点的基础上，逐步放宽放活有利于小城镇建设和发展的政策。改革小城镇土地使用和管理制度，促进土地配置方式的多样化。实行更加灵活的户籍制度，采取优惠的税费政策，扶持小城镇发展，鼓励单位和个人以多种方式参与小城镇的建设、经营和管理。三是加大对小城镇建设和发展的扶持力度。市财政安排一定数额资金，县级财政从城市建设维护税和公用事业附加中安排5％的资金，用于扶持小城镇的建设和发展。四是建立健全小城镇管理队伍，加大村屯环境综合整治力度，切实改善农村人居环境。

五、全面繁荣各项社会事业，维护社会稳定，努力构建和谐铁岭

增强社会保障能力，千方百计扩大就业。依法扩大各类社会保险覆盖面，提高各类保险的参保率。加大各类保险金的收缴力度，确保离退休人员养老金和享受失业保险待遇人员失业金100％社会化发放。就业和再就业是民生之本，要作为政府的“一号工程”。实施城乡就业统筹战略，完善基层劳动保障平台，逐步实现城乡劳动力双向自由流动。继续开发公益性岗位，千方百计增加就业岗位，力争为没有就业人员的家庭至少提供一人就业的机会。认真落实再就业的各项政策，调高和落实最低工资标准，加强就业培训，增强下岗失业人员的就业技能，全年组织培训1．73万人次。统筹各方面就业，继续做好复转军人安置工作。

继续加强科技和人才工作。实施科技创新工程，创办市高新技术创业服务中心，为发展高新技术企业提供条件，新增高新技术企业3户、民营科技企业70户，高新技术产业产值达到28亿元。坚持以信息化促进工业化，将陆平、北辰等10户企业列为市级制造业信息化应用示范企业。推广工厂化育苗、动植物病虫害防治等7项农业实用技术，建设5个农业科技示范基地，提高农产品的科技含量。加强对农民的教育和培训，提高农民的科技素质．积极探索建立多元化的科技投入体系，促进科技与经济的结合。进一步加强科技交流与合作，合作项目达到30个。继续加大人才工作力度，实施高层次人才引进、人才素质提升、人才环境建设“三大工程”，妥善安置大中专毕业生，全面实施“一村一名大学生”培养计划，今年再选调500名优秀农村青年脱产深造。

强化教育优先发展的战略地位。继续完善“以县为主”的教育管理体制，深入实施“春晓工程”，加强农村义务教育，积极推进课程改革。整合普通高中资源，改善办学条件，全市高中阶段扩招1500人，其中市本级扩招400人。大力发展职业教育、成人教育、民办教育，培养更多的实用型人才。加强高等学校专业建设，整合高等教育资源，采取设立分校、联合办学等形式，加快创办地方本科大学步伐。加强师资培训和职业道德建设，努力提高教育教学质量。

加快发展文化、卫生等各项社会事业。加强文化设施建设，建好图书馆暨少儿图书馆大楼，充实调整博物馆地志展内容。稳妥进行文化体制改革，大力发展文化产业，培育和打造以艺术节、小品小戏、绿色二人转、中国指画为重点的文化产业品牌，举办中国银冈书院文化研讨会，进一步推进文化名城建设。开展“扫黄打非”集中行动，净化文化市场。加强疾病预防控制、医疗救治，卫生监督执法“三个体系”建设，全面提升对重大群体性疾病、突发疫情的预警能力和公共卫生事件的应对能力。调整乡镇卫生院布局，集中力量办好20所乡镇中心卫生院和10所偏远地区乡镇卫生院，加强农村预防保健体系建设。改革农村卫生管理体制，抓好铁岭县建立农村新型合作医疗制度试点工作。强化社区卫生服务站的功能，努力提高医疗服务水平。加强医疗、医药行业管理，确保人民群众就医、用药安全。进一步加强广播电视工作。推进村村通工程，加快中心城区网络改造步伐并与各县(市)区联网。加快广播电视大厦工程建设，年底前竣工并投入使用。加强和改进广播电视宣传，提高舆论引导水平。广泛开展群众性体育活动，增加体育人口，构建多元化的全民健身服务体系。加强竞技体育，培养更多的体育人才，争取在省以上比赛中再创佳绩。建立并完善人口和计划生育工作的利益导向和社会保障机制，改革人口和计划生育管理体制和工作模式。开展传播新型生育文化、建设幸福小康家庭活动，不断提高计划生育服务水平。以社会公德、职业道德、家庭美德教育为重点，以城乡共建、创建文明城市等为载体，开展丰富多彩的精神文明建设活动。加强未成年人思想道德教育，清除不健康文化对青少年的侵蚀，为未成年人的健康成长创造良好的社会环境。继续做好复转军人安置工作。扎实开展“双拥”工作，进一步增进军政、军民团结。

协调好各方面利益关系，妥善处理各种社会矛盾。完善信访工作责任制，综合运用政策、法律、经济、行政手段和教育、协商、调节等方法，依法及时合理地处理群众反映的问题。进一步加大市民投诉问题的办理力度，确保市民投诉的问题在第一时间内得到妥善解决。加强城镇社区和农村自治组织建设，银州区完成55个社区建房任务，充分发挥社区和村委会在协调利益、化解矛盾、排忧解难方面的作用。继续开展扶贫帮困活动，认真落实城乡最低生活保障制度，建立困难群众大病救助和贫困母亲、贫困儿童救助制度，新建4所中心敬老院，开工建设残疾人康复培训中心，确保困难群体的基本生活。开展新一轮的“手拉手、共同致富”活动，促进少数民族地区经济的发展。

强化社会管理，维护社会稳定。推进平安县(市)区创建工作，夯实构建和谐社会的基础。深化公安管理体制和警务机制改革，开展经常性的严打活动，建立和完善社会治安防控体系，增强人民群众的安全感。扫除各种丑恶现象，净化社会环境。建立健全社会预警和应急机制，提高防范和处置突发事件的能力和水平。深入开展不稳定因素大排查、大调处工作，从源头上减少和化解矛盾纠纷。严格执行安全生产“三同时”制度，使安全生产逐步走上制度化、经常化、科学化轨道，从源头上预防安全事故。开展经常性的安全生产检查，认真落实事故隐患整改措施，避免重特大安全事故的发生。正确贯彻党的宗教政策，依法加强对宗教事务的管理，积极引导宗教与社会主义社会相适应。

六、切实加强政府自身建设，努力提高执政能力

面对新一年繁重的工作任务，我们必须按照十六届四中全会的要求，切实加强政府自身建设，努力提高各级政府的执政能力，为完成各项工作任务提供坚强的组织保证。

坚持科学行政、民主行政、依法行政。以科学的思想、科学的制度、科学的方法做好政府各项工作，通过多种渠道和形式广泛集中民智，成立由各方面专家组成的决策咨询委员会，建立重大决策失误责任追究制度和决策执行中的信息反馈、纠错改正机制，努力做到科学决策。自觉接受人大及其常委会的依法监督和人民政协的民主监督，认真执行人大关于政府工作的各项决议、决定，周密安排人大代表和政协委员的视察、检查活动，提高办理人大代表建议和政协提案的质量。加强同民主党派、工商联、无党派人士以及工会、共青团、妇联等人民团体的联系，认真听取各方面意见、建议并作为加强和改进政府工作的重要依据。坚持和完善政府领导与群众对话、重大事项新闻发布、涉及群众切身利益事项听证“三项制度”，不断拓宽政府与人民群众的沟通渠道，努力做到民主行政。认真贯彻《全面推进依法行政实施纲要》，进一步理顺行政执法体制。深入贯彻《行政许可法》，积极探索联合办理、集中办理行政许可的新机制。继续推行行政执法责任制和评议考核制，增强“两制”的严肃性和约束力。根据法律法规规定和经济社会发展需要，及时制定、修改和废止政府规范性文件。依法严肃认真地做好行政复议工作，坚决纠正违法和不当的具体行政行为，切实维护行政管理相对人的合法权利。切实加强行政执法队伍管理和业务培训，进一步加大行政执法监督力度，认真解决有法不依、执法不公和多头执法、执法扰民等群众反映强烈的问题，切实做到严格执法、公正执法和文明执法。

转变政府职能，改进工作方式。积极稳妥地推进行政管理体制和事业单位改革，加快政府职能转变步伐，开展第三轮行政审批制度改革，进一步削减审批事项，规范收费标准，逐步减少政府对企业经济行为的直接干预，把政府经济管理的职能尽快转到为市场主体服务和创造良好环境上来，努力建设服务型政府。全面推行政务公开，不涉及机密的政务要全部公开，增加政府工作的透明度，提高政府公信力。树立正确的政绩观，切实转变作风，坚决反对和大力纠正形式主义、官僚主义，进一步精简会议和文件。继续清理政府系统的各种表彰、奖励活动，大幅度地减少表彰奖励事项，对市政府各部门实行绩效考评的新办法。强化政务督查工作，政府确定的重要工作都要实行量化分解和目标管理，建立严格的承办工作责任制，确保各项工作落到实处。

着力抓好公务员队伍的素质建设，努力提高行政效能。在政府机关中全面扎实地开展以践行“三个代表”重要思想为主要内容的保持共产党员先进性教育活动，提高广大公务员的政治素质。扎实开展学习型机关创建活动，建立学习成果考核制度，把学习考核成果与公务员的提拔、奖惩结合起来，强化对学习的激励和约束机制，努力创建学习型机关，克服一部分公务员的知识恐慌和本领恐慌，使其成为各条战线的行家里手。有计划、有重点地搞好公务员的培训，创新培训方式，突出培训的实用性。继续选派公务员到国内外学习深造、到企业和基层挂职锻炼，使其开阔视野、增长才干。建立政府机关和公务员工作规范，制定服务标准，强化效能监察，切实增强工作责任心和使命感，确保政府机关高效运转。加强政府机关各级领导班子建设，增强团结，努力提高凝聚力、创新力和战斗力。

加强廉政勤政建设，把执政为民落到实处。严格执行中央有关廉洁自律的各项规定，认真贯彻《建立健全教育、制度、监督并重的惩治和预防腐败体系实施纲要》。要以解决群众反映强烈的突出问题为重点，坚决纠正征收征用土地、城镇房屋拆迁、企业违法排污、企业重组改制和破产中损害群众利益，以及拖欠农民工工资等问题。继续抓好治理教育乱收费、纠正医药购销和医疗服务中不正之风以及减轻农民负担工作。严肃查处领导机关和领导干部滥用权力、谋取非法利益的案件，利用人事权、司法权、审批权违法违纪的案件以及工程项目、土地管理、企业重组、产权交易、物资和服务采购等领域的违法违纪案件，坚决惩处腐败分子。坚持标本兼治，进一步建立健全各项廉政制度，从源头上预防和治理腐败。切实加强廉政和纪律教育，反对和制止奢侈浪费。广大政府公务员特别是各级领导干部，要廉洁自律，勤政为民，体察群众疾苦，倾听群众呼声，围绕群众最关心的问题，千方百计为群众办好事、办实事，切实做到权为民所用，情为民所系，利为民所谋，树立政府的良好形象。

各位代表，铁岭的发展已进入了关键时期，同时也面临着加快发展的最佳机遇。机不可失，时不我待。让我们紧密地团结在以胡锦涛同志为总书记的党中央周围，在中共铁岭市委的坚强领导下，开拓创新，埋头苦干，为早日实现强市富民的宏伟目标而努力奋斗！
